# Examining the impact of attentional focus and partner gaze on interpersonal coordination

**DOI:** 10.3758/s13423-025-02695-5

**Published:** 2025-04-23

**Authors:** M. C. Macpherson, A. J. Brown, L. K. Miles

**Affiliations:** 1 School of Psychological Science, University of Western Australia, Perth, WA Australia; 2 School of Psychological Sciences, Macquarie University, 16 University Avenue, Sydney, NSW 2113 Australia

**Keywords:** Interpersonal coordination, Gaze behavior, Social attention, Virtual reality

## Abstract

As a foundation of social interaction, interpersonal coordination is boosted in prosocial contexts but undermined by negative situations. Exactly how social factors shape coordination is, however, unknown. Previous literature demonstrates that for coordination to emerge people must attend to their interaction partners. This evidence, however, draws from sterile laboratory studies employing heavy-handed manipulations of little interpersonal relevance. By contrast, in more naturalistic contexts subtle differences in how people attend to themselves and others (e.g., a lingering glance vs. a suspicious side glance) can profoundly change the course of interaction. Understanding how social factors shape interpersonal coordination therefore requires consideration of aspects of attentional behaviour that better characterise everyday interaction. To this end, the current research employed virtual reality (VR) to explore how two core features of social attention, focus (self vs. other) and partner gaze (direct vs. averted), influence the spontaneous coordination of arm and head movements. The results indicated that: (i) coordination was enhanced in the other (cf. self) focus condition; and (ii) coordination was diminished in the averted (cf. direct) gaze condition. These findings provide novel evidence to indicate that the emergence of interpersonal coordination varies as a function of the everyday attentional behaviours that punctuate naturalistic social exchange. Of broad theoretical note, here we demonstrate that the among the factors constraining interpersonal coordination, the distribution of attention between self and other plays a meaningful role.

## Introduction

The dynamics of social interaction are tightly intertwined with the characteristics of social attention (Capozzi & Kingstone, 2023). A prolonged stare, or furtive glance, invite diverse interaction opportunities, affiliative and otherwise (Argyle & Cook, 1976). Chief among the social corollaries of attention is interpersonal coordination (Tognoli et al., 2020). As a foundation for social exchange, coordinating actions with others boosts prosocial behaviour and affiliation (Mogan et al., 2017; Rennung & Göritz, 2016). By contrast, contexts in which social connection is challenging are accompanied by reductions in coordination (e.g., conflict – Paxton & Dale, 2013; psychopathology – Macpherson et al., 2020) and associated interpersonal benefits. What is not known, however, is specifically *how* variation in social circumstances shapes the emergence of interpersonal coordination. Here we raise a novel proposition: coordination is shaped via *socially relevant* changes, that is those that are meaningful in naturalistic social contexts, in how people attend to others.

### Attention and coordination

The notion that social attention shapes interpersonal coordination is consistent with contemporary theory and research (Schmidt & Richardson, 2008). Guiding this work is an influential model of motor control, the Haken-Kelso-Bunz (HKB) equation (Haken et al., 1985) that serves to identify control parameters governing the emergence of coordination. Specifically, components of a system coordinate to the extent that they are: (i) moving at similar frequencies, and (ii) coupled – that is, there is potential for transfer of information between the components. Of note, coupling can be attentional whereby a perceptual link^1^ is sufficient to drive the spontaneous emergence of coordination (Schmidt et al., 1990). In this way, the HKB model indicates that factors that serve to strengthen or weaken attentional coupling also strengthen and weaken coordination.

Evidence for the link between attention and coordination is compelling (Repp & Su, 2013). A broad literature has documented systematic effects of variation in stimulus properties (e.g., amplitude, velocity; Snapp-Childs et al., 2011; Varlet et al., 2012) and perceptual strategies (e.g., anchoring, tracking; Roerdink et al., 2008; Schmidt et al., 2007) on coordination. A key theme to emerge from this work is that actions which serve to amplify the information required to successfully coordinate (e.g., motion of the target), enhance coordination. Making such information more salient strengthens coupling and bolsters coordination. Given attention modulates coordination, it is feasible that the social factors that shape interpersonal coordination do so by driving changes in attentional behaviour.

Empirical work exploring the link between attention and interpersonal coordination also provides robust evidence that the strength of the attentional coupling between people supports the emergence of coordinated behaviour (Schmidt & Richardson, 2008). Intentionally looking toward (or away from) others, strengthens (or weakens) coupling and resultant coordination (Richardson et al., 2007; Schmidt & O’Brien, 1997). On this basis it is viable that disruptions to coordination that accompany, for instance, argument (Paxton & Dale, 2013), norm violation (Miles et al., 2010), or impaired social functioning (Macpherson & Miles, 2023), result from individuals attentionally decoupling from others (e.g., by looking away or focusing attention inwardly) who are seen as quarrelsome, rude or threatening. However, evidence for this conjecture is scant. Coordination research that has considered social factors has not typically manipulated or measured attentional behaviour, while work focused on attentional coupling has rarely done so outside of minimally social contexts. Evidence that interpersonal coordination is shaped by socially relevant changes to attentional behaviour is needed to extend the extant literature.

### Attention and coordination in social contexts

Evaluating attention-coordination links during real-time social exchange is challenging. In everyday interactions, attentional patterns are meaningful and nuanced, serving diverse intra- and interpersonal functions. Gaze patterns, for instance, serve dual perceptual (e.g., information gathering; Lev-Ari et al., 2022) and communicative (e.g., social signaling; Argyle & Cook, 1976) roles that guide interpersonal processes and afford information exchange (Gobel et al., 2015). Similarly, head movements orient perceivers towards salient information *and* provide important nonverbal cues to others (Hietanen, 1999; Langton et al., 2000). To further complicate matters, attention may also be decoupled from the here and now and directed inwardly (e.g., mind wandering; Smallwood & Schooler, 2006). In this situation, gaze direction and head orientation lose utility as a means to understand the attentional focus or behavioural intentions of others. As such, studying single dimensions of social attention in isolation (e.g., gaze direction *or* attentional focus) is unlikely to represent the richness of naturalistic interpersonal exchange (Brunswik, 1956).

Consideration of the dual function of gaze in interpersonal coordination research has been limited. With respect to the perceptual (i.e., information gathering) role, while deliberately focusing gaze on, or away from, others results in corresponding changes in coupling strength (e.g., Richardson et al., 2007), little research has considered how the communicative functions of gaze direction impact interpersonal coordination. The gaze of others can have a powerful influence, not only by capturing an observer’s attention but also as a signal of affiliative intent (e.g., approach vs. withdrawal; Argyle & Cook, 1976), effects that may influence coupling, and in turn coordination. Indeed, related work shows that individuals match the kinematics of a partner more closely when directly observed by that person (Krishnan-Barman & Hamilton, 2019), while recent research reported preliminary evidence of an association between partner gaze and coordination. Using virtual reality (VR), Macpherson et al. (2024) demonstrated that averted (cf. direct) partner gaze decreased interpersonal coordination. We adopt the same method here.

## The current research

The current study explored whether patterns of social attention influence interpersonal coordination. We used VR to evaluate whether two core characteristics of attentional behaviour – focus and gaze – impact the extent to which people spontaneously coordinate actions during real-time interaction. To ensure naturalistic interpersonal behaviour, the procedure feigned a social interaction with a human-controlled avatar (i.e., another participant). In reality, the avatar was pre-programmed. Employing VR affords key advantages of precise control (e.g., when manipulating avatar gaze) and unobtrusive motion-tracking (e.g., for capturing coordination), while preserving essential features of social interaction (Zhao et al., 2015).

Across two virtual interactions, participants were instructed to direct their attention externally toward their interaction partner (other-focus condition), or internally towards themselves (self-focus condition). We also manipulated the attentional patterns of the interaction partner such that the participant was either the focus of their attention (direct-gaze condition) or not looked at (averted-gaze condition). During each interaction, we quantified coordination between the participant and avatar for two behaviours. First, to ensure comparability with previous research (e.g., Lumsden et al., 2014), we instructed participants to perform arm curls, and programmed the avatar to do the same (Macpherson et al., 2024). Second, taking an exploratory stance, we also captured participants’ spontaneous (i.e., uninstructed) head movements. Consistent with patterns of gaze behaviour, head movements serve multiple roles in interpersonal settings, affording perceptual exploration, and specifying key social information to others (Langton et al., 2000). For each behaviour, we estimated the extent to which participants’ movements were coordinated with those of the avatar. Via inbuilt eye tracking we also captured participant gaze patterns during each interaction.^2^

### Hypotheses

We anticipate that conditions which bolster attentional coupling will be associated with higher levels of spontaneous coordination. Specifically, we expect higher levels of arm movement coordination when participants focus their attention on their partner (*H1*: other > self), and when looked at by the avatar (*H2*: direct > averted). Further, we expect that the difference between arm-movement coordination in the direct and averted gaze will be greater when participants focus their attention on their partner as opposed to themselves (*H3*). When considering participant gaze, we expect that time spent looking at or near the avatar will increase coordination (*H4*), while more time spent looking around the virtual room (i.e., away from the avatar) will reduce coordination (*H5*). We expect a consistent pattern of effects for the exploratory analyses of head-movement coordination (*EH1–EH5*).

## Method

### Participants and design

The target sample size was determined a priori using G*Power (v 3.1.9.7; Faul et al., 2007). Macpherson et al. (2024) reported a large effect of partner gaze on coordination (f = 0.42); however, to our knowledge no research has explored the influence of self versus other focus in this domain. Taking a conservative stance, we used a smaller effect size (f = 0.25) to calculate the sample size required to detect an interaction between partner gaze and focus instructions. To provide 80% power to detect a significant mixed-effect interaction (α = .05) required a sample of 130 participants (65 per condition). To allow for incomplete data and exclusion of participants who did not believe the cover story, we set a stopping rule of 165 participants in total.

One hundred and sixty-four participants took part in the experiment. Ninety-five were recruited from an undergraduate participant pool and took part in exchange for course credit. The remaining 69 participants were recruited from a community sample and received a small monetary reimbursement. Only individuals aged 18 years and over with no injury or impairment that impacted their movements were eligible to take part. Data from 14 participants were excluded due to technical errors (*n* = 3), failure to follow task instructions (*n* = 2), or failure to believe the cover story (*n* = 9). This resulted in a final sample size of 150 participants (female = 96, male = 51, non-binary = 3; aged 18*–*53 years, *M* = 22.9 years, *SD* = 7.1 years).

The experiment employed a 2 (focus instruction: self vs. other) × 2 (avatar gaze: direct vs. averted) mixed design with repeated measures on the first factor. Participants were randomly assigned to avatar gaze condition. The research was reviewed and approved by the Human Research Ethics Committee at the University of Western Australia.

### Procedure and materials

Participants were recruited to a study examining how people attend to others in VR. They were told participation involved interacting with another person, first in VR where they both would be represented as avatars, and subsequently face-to-face. Upon arrival, participants were informed that the other person was completing the first part of the study in a nearby laboratory. In reality, there was no other person and therefore no face-to-face interaction. The cover story was included to give participants the understanding that they were engaging in a genuine social interaction with another person, albeit initially in a virtual context (for similar procedures, see Lumsden et al., 2012; Macpherson et al., 2024; Miles et al., 2011).

**Fig. 1:**
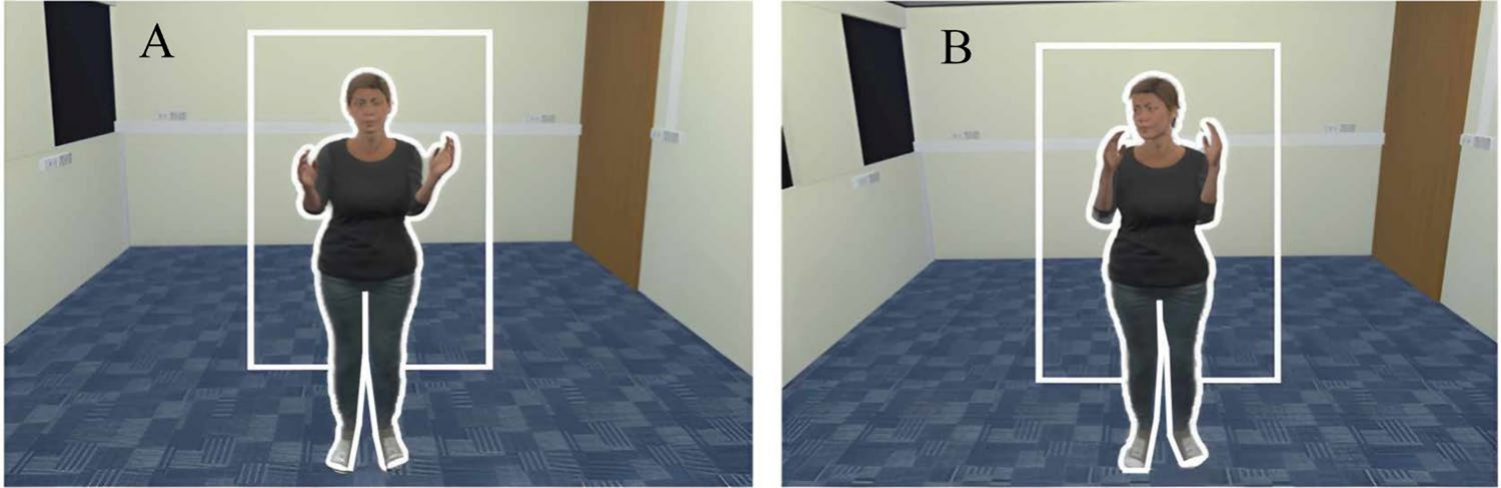
Direct (**A**) versus averted (**B**) avatar gaze during the interactive trials. The white outlines represent the areas of interest AOIs (i.e., at avatar, near avatar, around the room) used to categorise the participant gaze data

After providing consent, participants reported their age and gender in a free-response format.^3^ At this point, in order to enhance the believability of the cover story, the experimenter left the room to allegedly ‘check on the other participant’, returning a brief time later. Participants were then introduced to the VR system (Vive Pro Eye, HTC Corporation, Taiwan) and the virtual experimental environment (created using the Unity 3D Game Engine, v2018.4.8f1). To view the virtual environment, participants were fitted with a head-mounted display (HMD) equipped with dual OLED 3.5-in. screens (1,440 × 1,600 pixels per eye, 110° × 106° field of view) and in-built eye tracking. Eye tracking allowed estimates of the time participants spent looking at three pre-defined areas of interest (AOIs): (i) the avatar, (ii) near the avatar, and (iii) the rest of the room (see Fig. 1). To track participants’ arm movements, they were given two handheld controllers (Vive Pro, 2018). These recorded movement in three dimensions at a sampling rate of 50 Hz. In the VR environment, the controllers were represented as gloved hands.

Once in the virtual environment, participants completed a five-point eye-tracking calibration using the Super Reality (SR) runtime. They then completed a practice movement trial. Here, participants were placed in a generic grey virtual space and instructed to perform arm curls (flexion-extension about the elbow) in time with a metronome played through the HMD headphones (80 bpm, ≈ 1.33 Hz). Participants moved in time with the metronome for ten beats (approximately 7 s) after which they performed the arm curls without the accompanying metronome for a further 45 s. The practice trial was intended to familiarise the participant with the form and frequency of the movement required,^4^ as well as to give the experimenter an opportunity to make corrections if necessary (e.g., due to incorrect range of motion). Next, participants were placed in a virtual room designed to closely resemble a standard research laboratory (5.34 m × 4.34 m), where task instructions presented visually in the HMD informed them to “wait for the other participant to connect”.

During the short waiting period, participants were informed they would be completing two further VR arm-curl trials and both themselves and the other person would be represented as avatars. They were instructed to perform the same movement at the same tempo as they did during the practice trial. Instructions for the focus manipulation (self vs. other) were presented before the commencement of each trial via the HMD headphones. The instructions were adapted from McManus et al. (2008) and directed participants to focus on themselves [the other person] throughout the interaction by attending to their own [the other person’s] actions and monitoring how they [the other person] were coming across. Participants were reminded they would meet the other person in the next stage of the study. The order of focus instructions was counterbalanced across participants.

Participants interacted with an avatar that either looked directly at them (direct gaze condition, *n* = 79) or away from them (averted gaze condition, *n* = 71) for the duration of each trial. In the direct-gaze condition, the avatar was programmed to maintain eye contact with the participant while performing arm curls, whereas in the averted-gaze condition the avatar avoided eye contact, instead looking around the virtual laboratory. The avatar was created using Adobe Fuse CC (v 2017.1.0b) and rigged for movement using Adobe Mixamo (www.mixamo.com). The avatar was designed to resemble a typical university student in Australia (i.e., female, aged approximately 20–25 years, 1.64 m tall, casual clothing) and animated using arm-curl movements performed by an experimenter of similar stature and captured using a Rokoko Smartsuit Pro and Rokoko Studio (Rokoko, Copenhagen, Denmark). For the direct-gaze condition, the pre-recorded head movements were over-ridden using the animator controller in Unity3D, such that the head of the avatar followed the participant’s position. Each trial lasted for 45 s.^5^

After completing both trials participants were funnel debriefed to ascertain any suspicions they held regarding the cover story. Specifically, they were asked to report: (i) whether they had any particular goal or strategy while interacting with their partner, (ii) what they thought the VR task was trying to achieve, and (iii) whether anything seemed unusual about the VR task (see Macpherson et al., 2024, for a similar debriefing procedure). Those who indicated that they did not believe the cover story (*n* = 9) were excluded from the analyses. Participants were then debriefed as to the true purpose of the experiment and dismissed. Each testing session took no longer than 30 min.

### Data reduction and estimation of coordination

Consistent with previous work (e.g., Macpherson et al., 2020), to prepare the data for analysis, the first 5 s of each trial was discarded to avoid analysing any transient movement that may have occurred during the initiation of the arm curls. The remainder of the trial was then standardised to a length of 40 s. Next, each time series was centred around 0 and low pass filtered using a Butterworth filter with a 10-Hz cut-off.

To estimate coordination between participant and avatar arm movements, we calculated the relative phase relationship between the right arm of the participant and the left arm of the avatar,^6^ using a Hilbert transform. The resulting values were normalised to a range of 0–180° and the circular variance of the distribution of relative phase (rho) was calculated for each trial separately and standardised using a Fisher transformation. Rho provides a linear index of coordination stability whereby higher values represent more stable coordination.

Cross-recurrence quantification analysis (CRQA) was used to estimate coordination between participant and avatar head movements in the anterior-posterior plane.^7^ Estimation of the delay, embedding dimension, and radius parameters was performed using standard protocols (Coey et al., 2014). Using the avatar time series, the delay value was selected using the first minimum of the average mutual information, and the number of embedding dimensions was selected using the first minimum in a false nearest neighbour analysis. This resulted in a delay of 20 and an embedding dimension of 5. The radius was set to 12. As per previous literature (e.g., Macpherson & Miles, 2023; Romero et al., 2016), we then selected percent recurrence (%REC) as the outcome variable in our statistical analyses. %REC provides a measure of shared activity between interactants, whereby more coordination is indexed by higher values of %REC.

### Data analysis

To address the hypothesised effects, we constructed a series of mixed-effects models (MEMs) using the lme4 (Bates et al., 2015) and lmerTest (Kuznetsova et al., 2017) packages in R (v 4.3.0; R Core Team, 2019). For H1-H3 (and EH1–EH3), the models examined the influence of avatar gaze (direct vs. averted) and focus instruction (self vs. other), on the relevant coordination metric (arm movements: rho; head movements: %REC). For H4–H5 (and EH4–EH5), the percentage of time participants spent looking at each AOI (avatar, near avatar, room) was added separately as additional fixed effects.^8^

For each model, coding for factorial variables was as follows: focus instruction [1 = self, 2 = other], avatar gaze [1 = direct, 2 = averted], and all continuous predictor variables were centred prior to inclusion. Degrees of freedom and *p*-values were estimated using Satterthwaite approximations. The random effects structure for each model comprised a by-participant random intercept as this was the maximal model that would converge. Interaction effects were decomposed by estimating Tukey-corrected post hoc comparisons using the emmeans package (Lenth, 2021). After examining model fit, some of the models did not have normally distributed residuals. When this occurred, the relevant outcome variable was log-transformed.

When evaluating the exploratory hypotheses (i.e., EH1–EH5), we adopted a discovery-oriented stance (Tong, 2019; Tukey, 1977). That is, we did not disregard patterns evident within the results that offered insight to the effects of interest but did not meet traditional criteria for statistical significance (*p* <.05). This approach follows influential critiques that caution against blanket binary interpretation when using null hypothesis significance testing (e.g., De Groot, 1956/2014; Wagenmakers et al., 2012), and recognises that the calculation of *p*-values for mixed-effects models is not straightforward and presents complexities that other inferential tests do not (e.g., Kuznetsova et al., 2017). We acknowledge the caution required when taking this stance; however, given the strong theoretical grounds for predicting a role of visual coupling in interpersonal coordination (Schmidt & Richardson, 2008), we suggest this approach offers an appropriate examination of the current data.

## Results

### Arm-movement coordination (H1–H3)

**Fig. 2:**
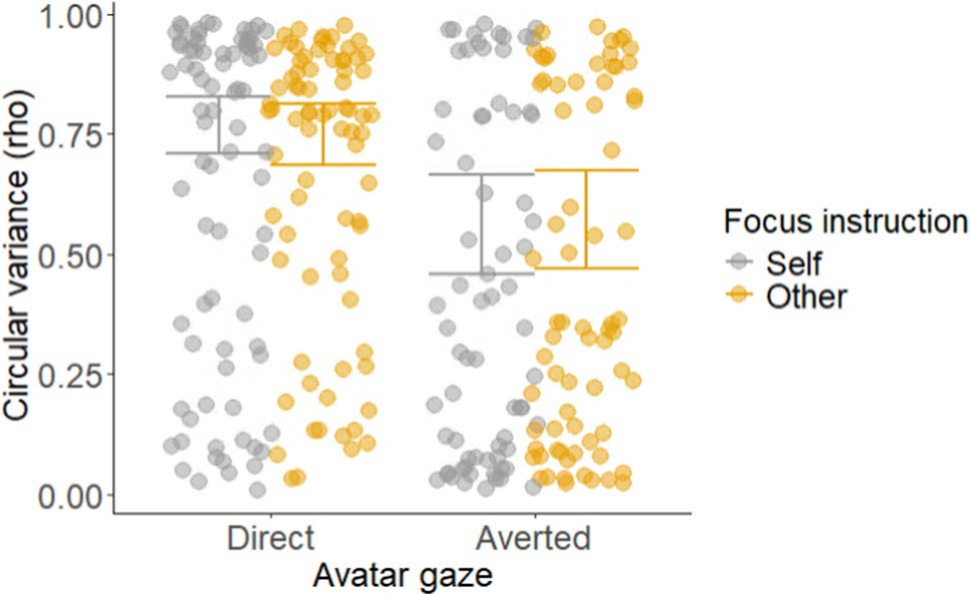
Arm movement coordination (rho) as a function of avatar gaze (direct vs. averted) and focus instruction (self vs. other). The raw (untransformed) data points for each participant are plotted alongside the model’s linear predictions to aid interpretation. Error bars represent 95% confidence intervals for the linear predictions

We first examined the effects of the manipulated variables on the coordination between participant and avatar arm movements (i.e., rho, Fig. 2). There was no main effect of focus instruction – coordination did not differ between the self and other-focus conditions, meaning we found no support for H1 (*b* = − 0.46, SE = 0.70,* t* = − 0.65, *p* =.52). There was, however, a main effect of avatar gaze. In support of H2, arm movements were more coordinated when the avatar looked directly at the participant than when gaze was averted (*b* = − 0.38, SE = 0.11,* t* = − 3.56, *p* <.001). There was no interaction between attention instruction and avatar gaze, and accordingly we found no support for H3 (*b* = 0.06, SE = 0.10,* t* = 0.59, *p* =.56).

### Head-movement coordination (EH1–EH3)

**Fig. 3:**
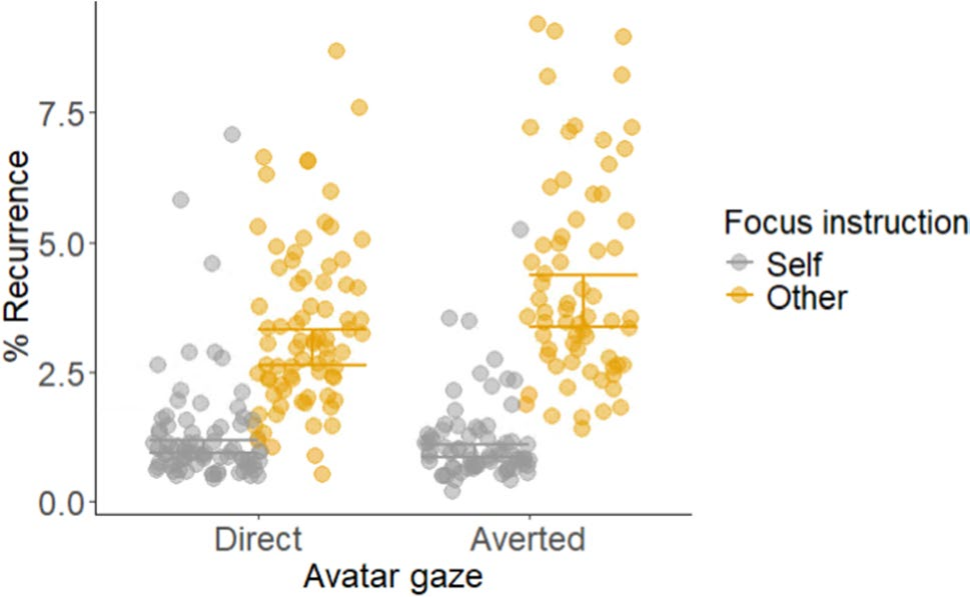
Head-movement coordination (%REC) as a function of focus instruction (self vs. other) and avatar gaze (direct vs. averted). The raw (untransformed) data points for each participant are plotted alongside the model’s linear predictions to aid interpretation. Error bars represent 95% confidence intervals for the linear predictions

When considering head-movement coordination (i.e., %REC), we found support for EH1 in terms of a main effect of focus instruction (Fig. 3). Here, greater coordination of head movements was observed in the other focus (cf. self focus) condition (*b* = 1.04, SE = 0.08,* t* = 12.68, *p* <.001). We did not find support for EH2, in that there was no main effect of avatar gaze (*b* = − 0.07, SE = 0.09,* t* = − 0.78, *p* =.44). There was, however, an interaction between focus instruction and avatar gaze (*b* = 0.33, SE = 0.12,* t* = 2.72, *p* = .007). Post hoc contrasts revealed higher levels of %REC when avatar gaze was averted, but only in the other focused condition (self: *b* = 0.07, SE = 0.09,* t* = 0.78, *p* = .86; other: *b* = -.26, SE = 0.09,* t* = − 2.96, *p* = .02). These results are counter to the effects predicted in EH3. That is, we observed higher levels of %REC when the avatar *looked away* from the participant, as opposed to toward.

### Participant gaze and coordination (H4–H5, EH4–EH5)

To examine the impact of participant gaze, we added each AOI (i.e., avatar, near avatar, room) separately to the models reported above and examined changes in model fit. For models with improved fit, we then examined the effects of participant gaze. For arm coordination, the addition of each AOI did not improve model fit (avatar: *χ*^*2*^(4) = 4.98, *p* = .29; near avatar: *χ*^*2*^(4) = 1.20, *p* = .88; room: *χ*^*2*^(4) = 6.06, *p* = .19).

For head coordination the addition of time spent looking at (*χ*^*2*^(4) = 12.47, *p* = .01), and near (*χ*^*2*^(4) = 11.60, *p* = .02), the avatar improved model fit. Each model revealed a two-way interaction between focus instruction and looking time (looking at: *b* = 1.46, SE = 0.54,* t* = 2.70, *p* = .008; looking near: *b* = − 1.88, SE = 0.65,* t* = − 2.91, *p* = .004). Post hoc tests revealed contrasting patterns in the relationship between %REC and participant gaze as a function of focus instruction, when considering both time spent looking at the avatar (self focus: *b* = − 0.51, SE = 0.27,* t* = − 1.92, *p* = .06; other focus: *b* = 0.49, SE = 0.35,* t* = 1.41, *p* = .16; contrast:* b* = − 1.00, SE = 0.43,* t* = *-*2.33, *p* = .02) and near the avatar (self focus: *b* = 0.68, SE = 0.37,* t* = 1.83, *p* = .07; other focus: *b* = − 0.98, SE = 0.60,* t* = − 1.64, *p* = .10; contrast: *b* = 1.65, SE = 0.69,* t* = 2.38, *p* = .02).

**Fig. 4:**
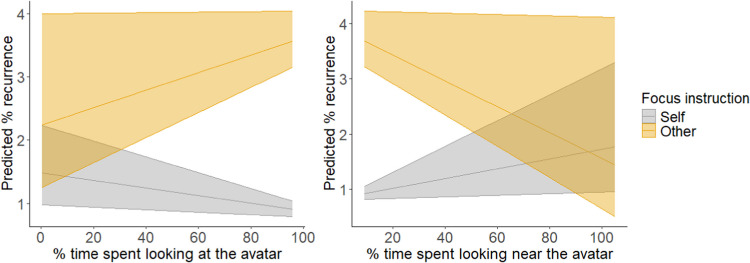
The relationship between head-movement coordination (predicted %REC) and the percent of each interaction spent looking at the avatar (**left panel**), and looking near the avatar (**right panel**), as a function of focus instruction (self vs. other). Predicted %REC is plotted as raw (untransformed) data to aid interpretation. The shaded area around the regression line represents the 95% confidence intervals for the linear predictions

Given that the slopes do not meet the traditional criteria for significance, the results offer no statistical support for EH4. However, given the strong theoretical grounds for a relationship between looking time and coordination, the observed trends do warrant consideration. For time spent looking *at* the avatar, participant gaze patterns showed a negative trend with coordination, but only in the self-focus condition. This trend was reversed in the other-focus condition. For time spent looking *near* the avatar, a contrary pattern was apparent – self focus showed a positive trend with coordination, while the other focus condition showed a negative trend (Fig. 4).

Finally, we did not uncover any effect of time spent looking around the room (*χ*^*2*^(4) = 3.64, *p* = .46), therefore providing no support for H5 or EH5.

## Discussion

This study investigated the influence of social attention on interpersonal coordination. In VR, participants performed a simple movement task with an avatar programmed to look at (direct gaze) or away from (averted gaze) them. Participants were instructed to direct their attention externally toward the avatar (other focus) or internally toward themselves (self focus). We quantified the degree to which participants coordinated their arm and head movements with those of the avatar. Several notable findings were revealed.

### Attentional focus and interpersonal coordination

As predicted, head-movement coordination was higher when participants were instructed to attend to the avatar. Focusing attention externally toward an interaction partner appeared to strengthen coupling and concomitant coordination in a manner consistent with actually looking toward their location (Richardson et al., 2007).^9^ While confirmation of the specific mechanism underlying this effect awaits future research, these results provide important evidence that a core property of everyday interaction – attending externally versus internally – shapes coordination. This indicates that the attentional factors constraining interpersonal coordination are not limited to perceptual couplings with the external environment – but extend to the effects of one’s mental focus.

Contrary to expectations, however, manipulating attentional focus did not impact arm coordination. We suspect this is due to the dynamic stability of the task. Performing simple rhythmic movements (e.g., arm curls) at an instructed frequency^10^ facilitates the emergence of stable interpersonal coordination that is resistant to perturbation (Schmidt & Richardson, 2008). In the current context, this stability may have limited the capacity for disruption to arm coordination by social and attentional factors (Macpherson & Miles, 2023; Macpherson et al., 2024). By contrast, it is possible that the patterns of variability intrinsic to uninstructed head movement were less resistant to perturbation, and therefore more likely to show the variation in incidental coordination observed here (Latash et al., 2002).

### Partner gaze and interpersonal coordination

Arm-movement coordination was more stable when the avatar looked at, compared to away from, participants. Replicating previous findings (Macpherson et al., 2024), this confirms that the gaze behaviour of others modulates the emergence of interpersonal coordination. This effect may stem from the fact that gaze direction specifies affiliative intent (Argyle & Cook, 1976). In the present study, direct gaze may have bolstered coordination by inviting engagement (Cui et al., 2019), while averted gaze, as a signal of disinterest (Capellini et al., 2019), may have encouraged disengagement (cf. Paxton & Dale, 2013). Insight as to precisely how presenting distinct affiliative opportunities shapes access to the information required for coordination is needed to confirm this account.

Although limited to one condition, the effect of partner gaze was contrary to predictions. When participants focused on the avatar, averted gaze led to *higher* levels of *head* coordination. While social exclusion (e.g., being ignored) often leads to withdrawal, it can also trigger ingratiating behaviour in an attempt to (re)connect (Williams, 2009). Here, participants who felt excluded may have been faced with mixed implicit goals, needing to balance withdrawal following exclusion, with the loss of social connection (Kashdan et al., 2008). We speculate that participants may have simultaneously pursued approach *and* avoidance goals via increased head coordination and decreased arm coordination, respectively. Given that increases in head coordination were only present when participants were directed to focus on the avatar, this may reflect attempts to catch their partner’s gaze. This possibility awaits further investigation.

### Participant gaze and interpersonal coordination

We found no evidence for a relationship between participant gaze and arm coordination. Again, we suspect that the simplicity of the arm curl task may play a role. In stable rhythmic tasks, interpersonal coordination is easily maintained via intermittent coupling, precluding the need for participants to consistently look at the avatar for coordination to emerge. Our exploratory analyses did, however, point to trends of interest when considering participant gaze and head coordination, although we caution against drawing firm conclusions as these effects did not meet the traditional criteria for significance. In the self-focus condition, the observed trends were suggestive of an association between coordination and the time spent looking at, as well as near, the avatar. However, when comparing between the models, the direction of these potential relationships differed. Although time spent looking *near* the avatar was associated with *improved* coordination, time spent looking *at* the avatar was associated with *decreased* coordination. Curiously, the opposite pattern was apparent for the other focus condition. As gaze direction can be decoupled from attentional focus, consideration of the psychological qualities that distinguish self- and other-focused attention (e.g., changes to self-awareness) would enable future research to better explore these potential relationships.

## Limitations and future directions

The current study examined coordination within a unidirectional context (i.e., participants coordinating with the avatar). However, coupling is frequently bidirectional, with coordination emerging via mutual information exchange between individuals. Extending the current work to consider dyadic interaction will not only allow for bidirectional coupling but would also permit coordination of naturalistic interpersonal gaze patterns to be quantified. Further, as noted, stability-related boundary conditions that constrain the influence of individual-level factors on coordination have been identified (Macpherson & Miles, 2023), and may also apply here. Future work should explore this possibility by scaling task stability (e.g., detuning; Schmidt & O’Brien, 1997) or complexity (e.g., conversation; Paxton & Dale, 2013) while capturing the effects of social attention. Finally, despite strong parallels between social behaviour and interpersonal coordination in and out of VR (Zhao et al., 2015), the contrivances of virtual environments mean it is important to explore the current effects in other contexts.

## Conclusion

Interpersonal coordination supports social exchange. Here, we demonstrated that two core characteristics of everyday social attention modulate the emergence of coordinated behaviour. Specifically, higher levels of coordination were associated with: (i) directing attention towards an interaction partner as opposed to the self, and (ii) being directly looked at by an interaction partner as opposed to their gaze being averted. Enacting social behaviour that serves to strengthen attentional coupling appears to be an effective strategy for enhancing interpersonal coordination.

## Data Availability

The data for this study is available on the Open Science Framework (https://osf.io/rhkt4/). The study was not preregistered.

## Footnotes

1 Attentional coupling can be made to extrapersonal (e.g., entrainment to an external rhythm), intrapersonal (e.g., bimanual coordination) or interpersonal (e.g., coordinating with others) sources of information.

2 We included this measure as an indication of spontaneous participant attentional patterns. However, given attention can be decoupled from looking direction, we acknowledge that interpretation of H4 and H5 must be tentative given our focus instruction manipulation.

3 As part of a separate project, participants also completed the Liebowitz Social Anxiety Scale (LSAS; Liebowitz, 1987) and the Social Behaviour Questionnaire (SBQ; Clark, 2005). These data are not reported here.

4 Participants were instructed to time their movements such that each metronome beep coincided with being at the top or bottom of the arm curl cycle (i.e., target movement frequency ≈ 0.667 Hz). In the experimental trials, the frequency of participant arm movements (0.680 Hz) was not appreciably different from this target, or the frequency of avatar arm movements (0.675 Hz). This indicates that participants correctly adopted the intended movement speed.

5 As a manipulation check, immediately after each trial we asked participants to rate where their attention was actually focussed (anchored by ‘only self’ and ‘only other’). Comparison between focus conditions indicated the manipulation was successful and participant ratings differed in the intended manner (self < other; *b* = 22.70, SE = 3.42,* t* = 6.63, *p* <.001), as did their actual gaze behaviour in terms of the time spent looking at the avatar (self < other; *b* = 0.16, SE = 0.04, *t* = 4.07, *p* <.001). We also asked for ratings of task difficulty (anchored by ‘not at all’ and ’very’) as research in non-social domains suggests cognitive processing may differ as a function of internal versus external focus (Dixon et al., 2014). Here participants reported finding the self-focus condition more difficult (*b* = - 12.80, SE = 3.46,* t* = - 3.70, *p* <.001). However, inclusion of difficulty ratings in the models predicting coordination (i.e., rho, %REC) did not improve the fit of either model, hence we did not further consider these ratings.

6 The opposite configuration (right arm of the avatar, left arm of the participant) revealed identical patterns of results and is therefore not reported here.

7 We focused on head movements in the anterior-posterior plane as these actions can carry social significance (e.g., proxemic behaviour) and, importantly, avatar head movements along this plane are identical across the averted and direct gaze conditions.

8 An additional five trials (from different participants) were excluded from the CRQA analyses (EH1–EH3) as the estimates for %REC were considered outliers (i.e., > 3 SD from the mean). Further, four participants were excluded from analyses that considered participant gaze patterns due eye-tracking errors.

9 As a preliminary evaluation of this claim, we examined patterns of participant gaze as a function of focus instruction. This indicated that when directed to focus on their partner, participants spent more time looking at the avatar, *b* = 0.16, SE = 0.04, *t* = 4.07, *p* <.001.

10 To ensure there were no inadvertent differences in movement frequency due to the experimental manipulations, we examined the mean and coefficient of variation (CoV) for both (i) arm-movement frequency, and (ii) head-movement frequency, as a function of partner gaze and focus instruction. There were no differences in the head-movement metrics based on the manipulations (*p* =.14 –.79), suggesting that the observed coordination differences cannot be explained by systematic changes in participants’ head-movement frequency or stability. Similarly, the experimental manipulations did not impact mean arm movement frequency (*p* =.22 –.99). Interestingly, focus instruction did impact CoV of arm movement frequency (*b* =.10, SE = 0.05,* t* = 2.14, *p* =.03), but this effect was not reflected in any measure of interpersonal coordination. These analyses effectively rule out differences in movement frequency as a potential explanation for any observed differences in coordination.

## Code availability

The code for this study is available Open Science Framework (https://osf.io/rhkt4/).
